# The effectiveness of chitosan as a hemostatic in dentistry in patients with antiplatelet/anticoagulant therapy: systematic review with meta-analysis

**DOI:** 10.1186/s12903-023-03568-w

**Published:** 2024-01-10

**Authors:** Giuseppe Minervini, Rocco Franco, Maria Maddalena Marrapodi, Marco Di Blasio, Marco Cicciù, Vincenzo Ronsivalle

**Affiliations:** 1 Saveetha Dental College and Hospitals, Saveetha Institute of Medical and Technical Sciences (SIMATS), Saveetha University, Chennai, Tamil Nadu India; 2 Multidisciplinary Department of Medical-Surgical and Dental Specialties, University of Campania Luigi Vanvitelli, Napoli, 81100 Italy; 3 Department of Biomedicine and Prevention, University of Rome “Tor Vergata”, Rome, 00100 Italy; 4 Department of Woman, Child and General and Specialist Surgery, University of Campania “Luigi Vanvitelli”, Naples, 80121 Italy; 5 Department of Medicine and Surgery, University Center of Dentistry, University of Parma, Via Gramsci 14, Parma, Province of Parma, 43126 Italy; 6 Department of Biomedical and Surgical and Biomedical Sciences, Catania University, Catania, 95123 Italy

**Keywords:** Chitosan, Bleeding, Oral Surgery, Hemostasis

## Abstract

Hemorrhage control is a crucial aspect of dental procedures, and achieving efficient hemostasis remains a key challenge. The advent of hemostatic dressings has revolutionized the field of dentistry by providing effective and convenient solutions for managing bleeding in vari-ous dental scenarios. This article aims to provide an overview of hemostatic dressings, their mechanisms of action, and their diverse applications in dentistry. We applied the following Pop-ulation, Exposure, Comparator, and Outcomes (PICO) model to assess the document eligibility. A literature search was performed on major search engines, using keywords. At the end of the search, 3 articles were selected that matched the PICO. Three items were selected after the screen-ing process, and bleeding times were analyzed between the control group and the study group. The overall effect showed a substantial and statistically significant difference with bleeding time in favour of HDD-treated patients, showing that this garrison is very useful in controlling bleed-ing for patients taking anticoagulants and antiplatelets (Mean difference − 5.61; C.I. -5.70, − 5.52); Overall, hemostatic dressings have revolutionized the management of bleeding in dentistry, offering a promising solution to achieve optimal hemostasis, improve treatment outcomes, and enhance patient care, particularly Hemcon.

## Introduction

Uncontrolled bleeding during dental procedures can compromise visibility, increase patient.

discomfort, prolong treatment time, and potentially lead to complications. Traditional methods like.

pressure, suturing, and the use of local hemostatic agents have limitations in certain clinical situations [1–3]. Bleeding, in the context of hemostasis, refers to the escape of blood from damaged blood vessels, a phenomenon that can be life-threatening if not properly controlled. To prevent uncontrolled bleeding, the human body employs a highly evolved coagulation system that rapidly forms blood clots, sealing the injured vessel and preventing the loss of blood. Coagulation, also known as blood clotting, is a tightly regulated process involving a cascade of biochemical reactions that ultimately lead to the formation of a stable, fibrin-rich clot at the site of injury.

This delicate equilibrium between bleeding and coagulation relies on an intricate interplay between vascular endothelial cells, platelets, and various coagulation factors found in the blood plasma. Dysregulation of this system can result in two major pathological conditions: hemorrhage, where blood fails to clot, and thrombosis, the unwanted formation of blood clots within intact blood vessels. Both conditions can have severe consequences for human health, leading to excessive bleeding or, conversely, to vascular occlusion and organ damage.

Hemostatic dressings have emerged as an alternative approach to achieving efficient hemostasis in dentistry, providing several advantages over conventional methods. Hemostatic dressings are materials designed to promote hemostasis by accelerating clot formation and preventing blood loss. They typically work through one or more of the following mechanisms:

- Absorption and swelling: Some dressings absorb blood and form a gel-like matrix, promoting platelet activation and aggregation.
- Hemostatic agents: Certain dressings contain active hemostatic agents like chitosan, kaolin, or oxidized cellulose, which facilitate clot formation.
- Adhesion and sealing: Dressings with adhesive properties can create a physical barrier over the wound, promoting clot stabilization and preventing further bleeding.
- Localized pressure: Some dressings, like sponge-based hemostats, apply pressure to the bleeding site, aiding in hemostasis.

Hemostatic dressings find diverse applications in various dental procedures. Extractions: Hemostatic dressings are utilized to control bleeding post-extraction, particularly in cases of patients on anticoagulant medications or with bleeding disorders. Periodontal procedures: Hemostatic dressings can aid in achieving hemostasis after scaling and root planing, periodontal surgery, or implant placement. Biopsy and oral surgery: Dressings are used to manage bleeding following soft tissue biopsies, frenectomies, or other oral surgical procedures [4–8]. Management of bleeding disorders: Hemostatic dressings offer an effective means of controlling bleeding in patients with clotting disorders or those undergoing dental procedures requiring anticoagulation therapy. In recent years, significant advancements have been made in hemostatic dressing technologies, leading to improved efficacy and ease of use [9–16]. These advancements include: (a) Nanotechnology-based dressings: Nanofabrication techniques have been employed to develop dressings with enhanced hemostatic properties, such as increased surface area for clotting and controlled release of hemostatic agents. (b) Biocompatible and biodegradable dressings: Researchers are focusing on developing dressings that are not only effective in achieving hemostasis but also biocompatible and biodegradable, reducing the risk of adverse reactions and promoting tissue healing. (c) Combination therapies: Hemostatic dressings are being combined with other therapeutic agents, such as growth factors or antimicrobial agents, to provide additional benefits and enhance treatment outcomes [17–19]. Straight chain cationic polysaccharide chitosan (deacetylated polysaccharide) is a naturally occurring or synthesized cationic copolymer made of 2-amino-2-deoxyglucose (60–100%) and 2-acetylamino-2-deoxyglucose-D-glucoside (0–50%). Although some fungi also contain chitin, it is mostly found in the exoskeletons of crustaceans. Rouget discovered chitosan in 1859 by treating chitin with a hot potassium hydroxide solution, which also established the framework for contemporary chitosan manufacture. After oral extraction treatments, chitosan works well as a wound dressing. After extractions on patients taking antithrombotic drugs continuously, a study evaluated the efficacy of chitosan-based dressings [20–25]. In extraction sites with chitosan dressing, the results showed no instances of dry sockets or pus discharge [26–31]. Chitosan dressing, a hemostatic agent, can dramatically lessen post-extraction bleeding and improve pain management in individuals taking continuous oral antithrombotic medicine. Due to its extended storage life, wide range of storage temperatures, and portability, powder hemostatic compounds have gained popularity in recent years. The purpose of this review with meta-analysis is to evaluate the possible use of HDD in the control of post-dental extraction bleeding in patients on antiplatelet-anticoagulant treatment.

## Materials and methods

### Eligibility criteria

We applied the following Population, Exposure, Comparator, and Outcomes (PICO) model [32] to assess the document eligibility:

P) Participants consisted of the population.

I) The Exposure consisted of patients who needed dental extractions and take anticoagulant or antiaggregant.

C) The comparison is made between patients treated with chitosan for hemorrhage control and patients treated with compression alone.
O) The outcome is to evaluate the possible impact of chitosan as a hemostatic agent in oral surgery.

Only studies providing data about the prevalence in both groups were included. We set the following exclusion criteria: (1) Patients with more than 3 missing teeth; (2) cognitive or intellectual disabilities (3) patients with cognitive and intellectual problems; (4) patients with partial removable dentures; (5) cross-over study design; (6) language different from English; (7) full- text unavailability (i.e., posters and conference abstracts); (8) studies involving animals; (9) review (topical or systematic) article; (10) case reports/series.

### Search strategy

We searched PubMed, Web of Science and Scopus for articles published from the inception until May 1, 2023. Table 1 is reported the search strategy used to search documents in the search engines.

**Table 1:** Search strategy

***PubMed*** hemostatic dressing AND dentistry (“haemostat“[All Fields] OR “haemostatically“[All Fields] OR “haemostatics“[All Fields] OR “hemostatics“[Pharmacological Action] OR “hemostatics“[MeSH Terms] OR “hemostatics“[All Fields] OR “haemostats“[All Fields] OR “hemostasis“[MeSH Terms] OR “hemostasis“[All Fields] OR “haemostatic“[All Fields] OR “hemostat“[All Fields] OR “hemostatically“[All Fields] OR “hemostatic“[All Fields] OR “hemostats“[All Fields]) AND (“bandages“[MeSH Terms] OR “bandages“[All Fields] OR “dressing“[All Fields] OR “dressings“[All Fields] OR “dress“[All Fields] OR “dressed“[All Fields] OR “dresses“[All Fields] OR “dressing s“[All Fields]) AND (“dentistry“[MeSH Terms] OR “dentistry“[All Fields] OR “dentistry s“[All Fields])
***Web of Science*** (hemostatic dressing) AND (dentistry) (ALL FIELDS) ***Scopus***
TITLE (hemostatic dressing) AND (dentistry) ***Cochrane*** (hemostatic dressing) AND (dentistry) (ALL FIELDS) ***NCBI*** hemostatic dressing AND dentistry (“haemostat“[All Fields] OR “haemostatically“[All Fields] OR “haemostatics“[All Fields] OR “hemostatics“[Pharmacological Action] OR “hemostatics“[MeSH Terms] OR “hemostatics“[All Fields] OR “haemostats“[All Fields] OR “hemostasis“[MeSH Terms] OR “hemostasis“[All Fields] OR “haemostatic“[All Fields] OR “hemostat“[All Fields] OR “hemostatically“[All Fields] OR “hemostatic“[All Fields] OR “hemostats“[All Fields]) AND (“bandages“[MeSH Terms] OR “bandages“[All Fields] OR “dressing“[All Fields] OR “dressings“[All Fields] OR “dress“[All Fields] OR “dressed“[All Fields] OR “dresses“[All Fields] OR “dressing s“[All Fields]) AND (“dentistry“[MeSH Terms] OR “dentistry“[All Fields] OR “dentistry s“[All Fields])

We also manually searched published systematic and topical reviews on similar topics.

We conducted this systematic review following the guidance of the Cochrane Handbook for Systematic Reviews of Interventions and the Preferred Reporting Items for Systematic Reviews (PRISMA) guidelines 2020. The systematic review protocol has been registered on the International Prospective Register of Systematic Reviews (PROSPERO) with CRD42022327470.

### Data extraction

The data were extracted manually, reviewing each source and selecting relevant information. Extracted data were reported on a Microsoft Excel sheet and were evaluated independently by two reviewers (MC and RF). In disagreement, a consensus was reached through a third reviewer (MDA). The following data were extracted: (1) First author; (2) Year of publication; (3) Nationality; (4) Type of study; (5) Number of participants; (6) Type of drugs; (7) Bleeding time between two groups; (8) Significance of the study.

### Quality assessment

The Version 2 of the Cochrane risk-of-bias tool for randomized trials (RoB 2) were used by two reviewers (MC and GM) to assess the risk of bias in the included studies. The Cochrane RoB 2 tool is a well-established tool for evaluating the quality of randomized trials. It considers six domains of potential bias: random sequence generation, allocation concealment, blinding of participants and personnel, blinding of outcome assessment, incomplete outcome data, and selective reporting. Any disagreement was discussed until a consensus was reached with a third reviewer (MDB).

### Grade of strenght

We applied the Grading of Recommendations Assessment, Development and Evaluation (GRADE) ranking system to measure the quality of evidence and also to determine the level of certainty for the results of this review.

### Statistical analysis

The software Review Manager version 5.2.8 (Cochrane Collaboration, Copenhagen, Denmark; 2014) was used to perform the pooled analysis. We measured the risk ratio (RR) on the ‘occurrence of complications between the two groups (HDD vs Compressive) treated with extractions. The Higgins Index (*I2*) and the chi-square test were implemented to assess Heterogeneity among studies. We classified heterogeneity as follows: low heterogeneity (< 30%), medium heterogeneity (30–60%), and high heterogeneity (> 60%).

## Results

### Study characteristics

**Fig. 1:**
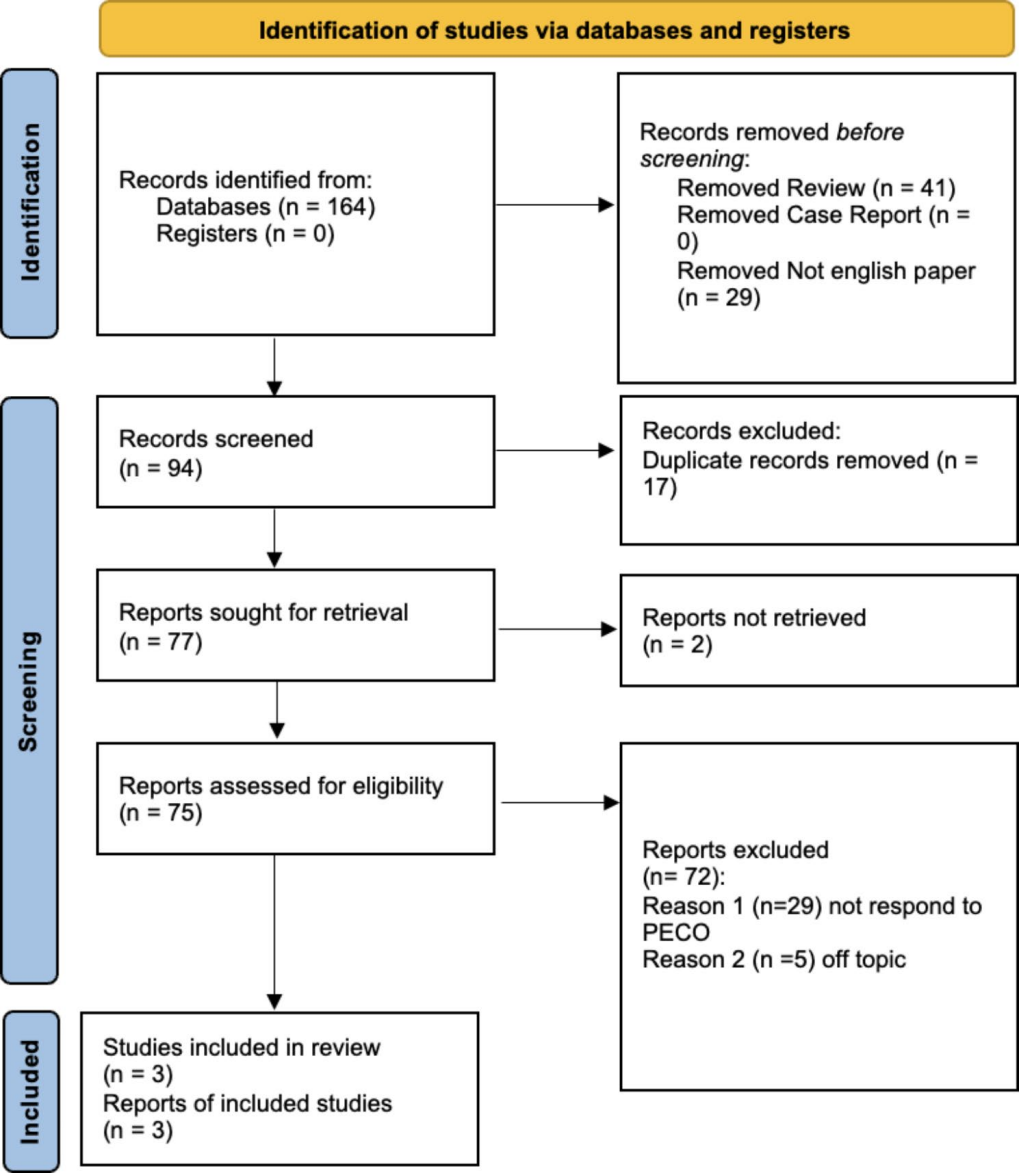
Prisma flowchart; *From*: Page MJ, McKenzie JE, Bossuyt PM, Boutron I, Hoffmann TC, Mulrow CD, et al. The PRISMA 2020 statement: an updated guideline for reporting systematic reviews. BMJ 2021;372:n71. 10.1136/bmj.n71 For more information, visit: http://www.prisma-statement.org/

Three studies were included in the systematic review and were considered for the metanalysis, as illustrated in the PRISMA 2020 flowchart in Fig. 1. 164 articles were selected because of the search. Seventy papers were excluded before the screening: 41 articles were not in English, and 39 were reviewed. The remaining 94 articles were selected for the title and abstract screening to evaluate whether they met the PECO criteria. 17 records were duplicates and, therefore, were excluded. Seventy-seven articles were assessed for eligibility. Among these, two were not retrieved, 25 were excluded because don’t respond to the PECO questions because they assessed bleeding not in dental extractions. The included studies have been published between 2012 and 2023. The seven included studies were retrospective cohort studies in design or randomized clinical trials. All these studies compare bleeding time in patients taking anticoagulants/antiplathelet evaluating bleeding times. The data extracted from each study, as reported in the paragraph “data extraction,” were written in Table 2.

**Table 2:** Principal elements of the studies which formed part of the present systematic analysis;

Author	Year	Nationality	Type of study	Number of teeth extracted	Complications with bruxism and no bruxism	Clinical significance
Pippi	2013	Italy	Split mouth randomized controlled trial	20 with HDD 20 control	Time Hemostasis: Control:7.58 min Study: 4.7 min	Chitosan has statistically significant effect on bleeding
Schmitter	2023	Germany	Split mouth randomized controlled trial	74 HDD 52 control	Time Hemostasis: Control: 9.53 min Study: 0.50 min	Chitosan has statistically significant effect on bleeding
Kumar	2016	India	Split mouth randomized controlled trial	33 HDD 33 control	Time Hemostasis: Control: 4.06 min Study: 1.49 min	Chitosan has statistically significant effect on bleeding
Sharma	2017	India	Split mouth randomized controlled trial	40 HDD 40 Control	Time Hemostasis: Control: 14.1 min Study: 1.13 min	Chitosan has statistically significant effect on bleeding
Kale	2012	Saudi Arabia	Split mouth randomized controlled trial	40 HDD 40 Control	Time Hemostasis: Control: 153 s min Study: 918 s	Chitosan has statistically significant effect on bleeding
Radhakrishna	2022	India	Split mouth randomized controlled trial	54 HDD 54 Control	Time Hemostasis: Control: 796 s Study: 96 s	Chitosan has statistically significant effect on bleeding
Sarkar	2022	India	Split mouth randomized controlled trial	30 HDD 30 Control	Time Hemostasis: Control: 158 s Study: 70 s	Chitosan has statistically significant effect on bleeding

**Table 3:** The grade summary of evidence

Certainty assessment							Summary of findings				
Participants (studies) Follow-up	Risk of bias	Inconsistency	Indirectness	Imprecision	Publication bias	Overall certainty of evidence	Study event rates (%)		Relative effect (95% CI)	Anticipated absolute effects	
							With compressive gauze	With HDD		Risk with compressive gauze	Risk difference with HDD
**HDD vs. Compressive (follow-up: range 1 days to 1 days; assessed with: Time; Scale from: 0.00 to 15)**											
680 (7 RCTs)	not serious	not serious	not serious	not serious	very strong association	⨁⨁⨁⨁ High	318	362			SMD **3.22 SD lower** (3.53 lower to 2.9 lower)

**CI**: confidence interval; **SMD**: standardised mean difference

### Main findings

Pippi’s study evaluated the efficacy of HemCon® Dental Dressing (HDD, Zimmer Holdings; HemCon Medical Technologies, Inc.,Beaverton, OR), a kiosan bse component used by the armed forces during the war in Iraq and Afghanistan. The study was performed at Umberto I Hospital and evaluated patients on antiplatelet therapy. Twenty patients were enrolled who were given two extractions at opposite sites. Product was applied at the study site while a collagen sponge was inserted at the control site. Patients were monitored for one hour and the bleeding time was set after 30 s that stopped. The mean bleeding time (T2) in the control group (282.15 ± 235.89s; range 66-723s) was significantly lower than in the test group (455.40 ± 418.33s; range 57-2216s); this difference is statistically significant (p = 0.0278) [33].

Malmquist’s study evaluated the effects of HemCon dental dressing as an antihemorrhagic following dental surgery. Patients were selected with the following inclusion criteria: taking oral antiplatelets; need for at least two dental extractions. Seventy-four sites treated with HemCon and 52 control were evaluated. After that, the examiner assessed bleeding times. All 74 HDD study sites, including 9 patients taking OAT, achieved hemostasis in less than 1 min while the control site showed a bleeding time of 9.53 min [34].

Kumar’s study evaluated the ability of Hemcon dental dressing for hemostasis on post-extractive dental alveoli.The study was structured as a double split mouth. Inclusion criteria were presence of at least two dental elements to be extracted; taking oral anticoagulants. Therefore, Hemcon was applied at the study site and compressive gauze was applied at the control site. There were 33 patients recruited of which 33 in 33 sites were study and 33 were control. The recruited patients did not discontinue their anticoagulant and had their INR below 4. Using HDD took less time to reach hemostasis than using a pressure pack did. For the control site, hemostasis took an average of 4.06 min to achieve. Hemostasis at HDD sites was shown to be statistically significant [35].

Sharma’s study evaluated the efficacy of chitosan as a hemostatic agent in a group of patients on antiplatelet therapy. The study was designed as a double blind a splith mouth. Forty patients on antiplatelet therapy for various cardiological reasons were recruited. Therefore, 80 extractions were performed and chitosan was applied at the study site, and compression was performed in the control group. The mean time for hemostasis at the study site was 1.13 s while in the control group it was 14 min, thus a statistically significant result [36].

Kale’s study evaluated the efficacy of chitosan as a hemostatic agent in a group of patients on antiplatelet therapy. The study was designed as a double blind a splith mouth. Forty patients on antiplatelet therapy for various cardiological reasons were recruited. Therefore, 80 extractions were performed and chitosan was applied at the study site, and compression was performed in the control group. The mean time for hemostasis at the study site was 53 s while in the control group it was 918 s, thus a statistically significant result [37].

Radhakrishna’s study evaluated the efficacy of chitosan as a hemostatic agent in a group of patients on antiplatelet therapy, in this case both single and double. The study was designed as a double blind a splith mouth. In addition to assessing bleeding time, site healing was evaluated. Fifty-four patients on antiplatelet therapy for various cardiologic reasons were recruited. Therefore, 108 extractions were performed and chitosan was applied at the study site and compression was performed in the control group. The mean time to hemostasis at the study site was 90 s while in the control group it was 109 s, thus a statistically significant result [38].

Sarkar’s study evaluated the efficacy of chitosan as a hemostatic agent in a group of patients on antiplatelet therapy, in this case both single and double. The study was designed as a double blind a splith mouth. In addition to assessing bleeding time, site healing was evaluated. Sixty patients on antiplatelet therapy for various cardiologic reasons were recruited. Therefore, 30 extractions were performed and chitosan was applied at the study site and PRF was applied in the control group for hemostatic purposes. The mean time to hemostasis at the study site was 70 s while in the control group it was 158 s, thus a statistically significant result [39].

### Meta-analysis

The included studies had a high heterogeneity (*I*2 = 99%). Therefore the meta-analysis was conducted by random model effect and continuous otucomes. We consider bleeding time as a variable and standard deviation and compare it between sites that received HDD and surgical sites controlled with simple compression. Kumar’s study is the only one that recruited patients treated with anticoagulants.

**Fig. 2:**
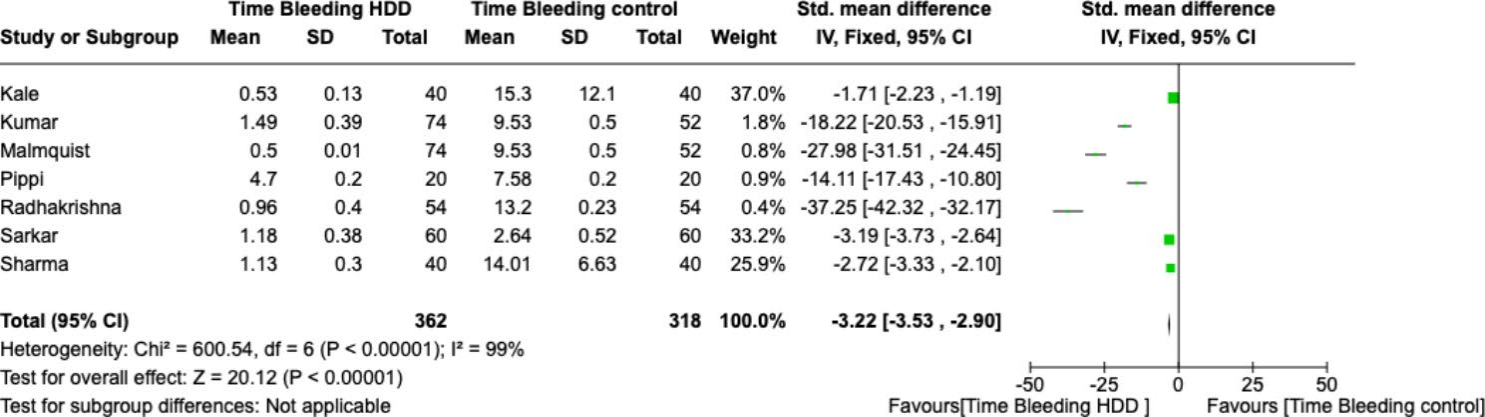
Forest plot of the meta-analysis

The overall effect, reported in the forest plot (Fig. 2), showed a substantial and statistically significant difference with bleeding time in favour of HDD-treated patients, showing that this garrison is very useful in controlling bleeding for patients taking anticoagulants and antiplatelets (Mean difference − 3.22; C.I. -3.53, − 2.90). The GRADE analysis showed a high degree of evidence for the results of the meta-analysis (Table 3).

### Quality assessment and risk of bias

**Fig. 3:**
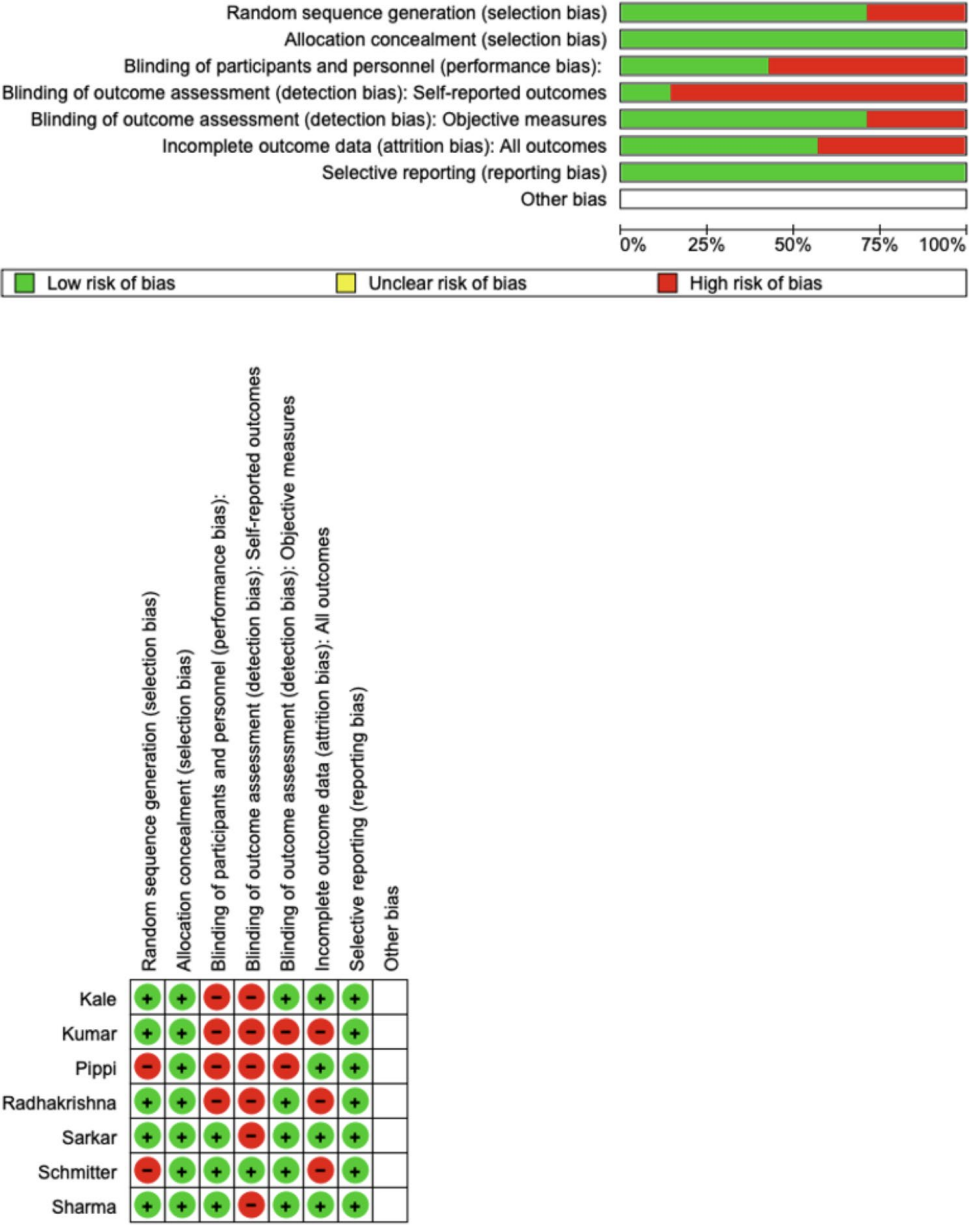
Risk-of-bias domains of included studies

The risk of bias in the included studies was reported in Fig. 3. Regarding the randomization process and allocation concealment, 25% had a low risk of bias. All studies excluded a performance; two study ensured an increased risk of detection bias (self-reported outcomes), and 2 of the included studies present low detection bias (Fig. 3). GRADE Table 3 shows a high level of evidence. This, however despite the heterogeneity of the studies, the results of the meta-analysis are evident and therefore leave no doubt about the evidence that HDD can be an aid to hemostasis.

## Discussion

The continuous advancements in hemostatic dressing technologies hold great promise for the future of dentistry [40–42]. Further research and development are required to optimize the properties and performance of hemostatic dressings in dental applications. Some potential future directions include:

- Customized formulations: Tailoring hemostatic dressings to meet specific dental needs, such as varying tissue types, oral environment conditions, and bleeding profiles, can enhance their efficacy and versatility.
- Controlled release systems: Incorporating controlled release mechanisms within dressings can enable the sustained delivery of therapeutic agents, such as antiseptics or growth factors, to enhance wound healing and reduce the risk of postoperative complications.
- Bioactive dressings: Integration of bioactive components, such as peptides or growth factors, into hemostatic dressings can promote tissue regeneration, reduce inflammation, and expedite the healing process in dental wounds.
- Enhanced tissue integration: Improving the interaction between dressings and the surrounding tissues can facilitate better wound healing and minimize the risk of dislodgement or infection. Strategies such as surface modifications or bioadhesive properties can enhance tissue adhesion and integration.
- Clinical guidelines and protocols: Developing standardized guidelines and protocols for the use of hemostatic dressings in dental procedures can promote consistent and optimal utilization, ensuring patient safety and predictable outcomes.
- Cost-effectiveness analysis: Conducting cost-effectiveness studies to evaluate the economic impact of incorporating hemostatic dressings into dental practice can provide valuable insights for healthcare providers and policymakers, facilitating informed decision-making.

Hemostatic dressings have revolutionized the management of bleeding in dentistry, offering effective and convenient solutions in various clinical scenarios. Through their diverse mechanisms of action and applications, these dressings have improved treatment outcomes, patient comfort, and procedure efficiency. Advancements in hemostatic dressing technologies, such as nanotechnology-based formulations and combination therapies, hold great promise for the future of dentistry. Continued research and development efforts are crucial to optimizing the properties, efficacy, and cost-effectiveness of these dressings, further improving their clinical utility and patient outcomes in the dental field. Acknowledging the importance of evidence-based practice, further research is needed to establish the clinical effectiveness of hemostatic dressings in different dental procedures. Rigorous clinical trials comparing hemostatic dressings with conventional methods and evaluating their long-term outcomes, patient satisfaction, and potential complications will contribute to the growing body of evidence.

Moreover, educating dental professionals on the appropriate selection and utilization of hemostatic dressings is essential. Training programs and continuing education courses can enhance dentists’ understanding of the available dressing options, their indications, and proper application techniques. This will ensure the optimal use of hemostatic dressings and promote their integration into routine dental practice [10]. The hydrophilic polysaccharide chitosan has a multitude of uses in biodental due to its antibacterial, immunostimulatory, hemostatic, and wound-healing characteristics [43]. Chitosan is used in pediatric dentistry to stop cariogenic germs from adhering to the mucosa. Because of its antibacterial and antiplaque properties, it is used in conjunction with mouthwash and chewing gum. Chitosan is used in endodontics to promote hemostasis for pulpotomy and is combined with glass ionomer cement for regenerative endodontics and dental bonding systems.

Chitosan that has been freeze-dried and then molded into a highly electropositive sponge-like substance is used to create HemCon [44, 45]. Due to its ability to connect with red blood cells, this charge makes it easier for blood clots to develop. Red blood cells, which are negatively charged, connect to the electropositive Hem-Con Bandage material, creating a very viscous clot that forms quickly and produces hemostasis by sealing the wound site. Shen et al. [46] demonstrated that exposure to chitosan enhanced the release of growth factor from human platelets, which could assist to explain our encouraging results. According to Cunha-Reis et al.‘s [47]findings, the HDD material utilized in this investigation exhibits cell adhesion. Comparing the HDD-treated sites to the control sites, the HDD-treated sites saw better postoperative healing with fewer problems. This could be linked to chitosan’s antibacterial characteristics, which have been studied in vitro experiments. The findings showed that chitosan made the inner and outer membranes more permeable and, in the end, broke down the bacterial cell membranes, allowing their contents to escape. Thus, HDD acts as an antibacterial barrier against a variety of Gram positive and Gram negative microorganisms, such as Acinetobacter baumannii, vancomycin-resistant enterococcus (VRE), and methicillin-resistant Staphylococcus aureus (MRSA).Azargoon et al. discovered that HDD is just as effective as ferric sulfate in promoting haemostasis and wound healing in an animal research [48–53].

The electrostatic attraction of RBC to the HemCon substance is what makes HDD self-adhesive .

In conclusion, hemostatic dressings have transformed the field of dentistry by providing effective and convenient solutions for managing bleeding during various dental procedures. Their diverse mechanisms of action and applications make them invaluable tools in achieving hemostasis, particularly in challenging clinical scenarios. With ongoing advancements in technology and increasing research focus, hemostatic dressings hold great promise for further improving patient care, enhancing treatment outcomes, and advancing the field of dentistry [54–57].

In conclusion, hemostatic dressings have emerged as a significant advancement in the field of dentistry, offering efficient and convenient solutions for achieving hemostasis during various dental procedures. Traditional methods have limitations in terms of efficacy and ease of use, making hemostatic dressings a valuable addition to dental practice. Their mechanisms of action, including absorption and swelling, hemostatic agents, adhesion and sealing, and localized pressure, provide effective means of controlling bleeding.

Hemostatic dressings find diverse applications in dentistry, ranging from extractions and periodontal procedures to biopsy and oral surgery. They are particularly useful in cases involving patients on anticoagulant medications or with bleeding disorders, where achieving hemostasis is challenging. Hemostatic dressings not only promote clot formation but also provide additional benefits such as improved tissue integration and controlled release of therapeutic agents [58–61].

Recent advancements in hemostatic dressing technologies, such as nanotechnology-based formulations and combination therapies, hold promise for further improving their efficacy and versatility. Customized formulations, controlled release systems, and bioactive dressings are among the potential future directions that can enhance the performance of hemostatic dressings in dental applications [62, 63] .

To fully realize the potential of hemostatic dressings, ongoing research efforts, clinical trials, and the establishment of standardized guidelines are necessary. Further studies should focus on evaluating the clinical effectiveness, long-term outcomes, patient satisfaction, and potential complications associated with hemostatic dressings in different dental procedures.

Education and training programs for dental professionals are crucial to ensure their proper selection and utilization of hemostatic dressings. By increasing awareness and understanding of available options, indications, and application techniques, dentists can maximize the benefits of hemostatic dressings and integrate them seamlessly into routine dental practice [64–66].

In summary, hemostatic dressings have revolutionized the management of bleeding in dentistry, offering effective solutions and improving treatment outcomes. With ongoing advancements in technology and increased research focus, hemostatic dressings hold immense potential to further enhance patient care, optimize treatment outcomes, and shape the future of dentistry.

Sarkar’s study unlike the other studies, uses PRF as the control group and this could be a confounding factor. However, other studies, considered for the meta-analysis, also failed to analyze and differentiate patients on single or dual antiplatelet. Another factor that could alter the results. The result of our meta-analysis, despite the heterogeneity of the studies, is in favor of an antihemorrhagic effect of chitosan compared with both PRF and compression gauze.

## Conclusions

In conclusion, hemostatic dressings have emerged as valuable adjuncts in dentistry, offering effective and efficient control of bleeding during various dental procedures. The different types of hemostatic dressings, including oxidized cellulose, gelatin-based, chitosan, and calcium-based dressings, provide localized hemostasis, reduce chair time, and improve patient outcomes.

The versatility of hemostatic dressings allows their application in a wide range of dental scenarios, including tooth extractions, periodontal surgery, implant placement, and biopsies. They are particularly beneficial for patients with bleeding disorders or those taking anticoagulant medications, as they provide a localized hemostatic effect while minimizing systemic risks.

The benefits of using hemostatic dressings in dentistry include enhanced visibility and control for the clinician, reduced chair time, and a decreased risk of postoperative complications such as bleeding and hematoma formation. By facilitating faster clot formation and promoting uneventful wound healing, hemostatic dressings contribute to improved patient comfort and satisfaction.

It is important for dental professionals to stay updated with the latest advancements in hemostatic dressings and understand their appropriate application techniques. Further research and clinical studies are warranted to explore the long-term efficacy, cost-effectiveness, and potential complications associated with different types of hemostatic dressings in dentistry.

Overall, hemostatic dressings have revolutionized the management of bleeding in dentistry, offering a promising solution to achieve optimal hemostasis, improve treatment outcomes, and enhance patient care.

## Data Availability

The data will be available on reasonable request from the corresponding author.

## References

[1] Burkatovskaya M, Castano AP, Demidova-Rice TN, Tegos GP, Hamblin MR. Effect of chitosan acetate bandage on wound healing in infected and noninfected wounds in mice. Wound Repair and Regeneration. 2008;16(3):425–31. Available from: https://www.scopus.com/record/display.uri?eid=2-s2.0-43349107758&=10.1111%2fj.1524-475X.2008.00382.x&=inward%3C=083789cad69115de726ab656565aa95b. 10.1111/j.1524-475X.2008.00382.x PMC2805166 18471261

[2] Alam HB, Chen Z, Jaskille A, Querol RILC, Koustova E, Inocencio R et al. Application of a zeolite hemostatic agent achieves 100% survival in a lethal model of complex groin injury in swine. Journal of Trauma - Injury, Infection and Critical Care. 2004;56(5):974–83. Available from: https://www.scopus.com/inward/record.uri?eid=2-s2.0-2942705909&=10.10972f01.TA.0000127763.90890.31&partnerID=40md5=772714eb297e047becbb3efba05c0ebb. 10.1097/01.ta.0000127763.90890.31 15179235

[3] McCarthy SJ, Teach J, Buckley L, Qian RQ, Gibson L, Lucchesi L et al. A novel chitosan dressing. Hemostatic efficacy in acute swine injury models: Splenic stripping compared to carotid laceration. In: Transactions – 7th World Biomaterials Congress. 2004. p. 349. Available from: https://www.scopus.com/inward/record.uri?eid=2-s2.013844299537&partnerID=40&md5=b8155fd53637d0ef31988dbf5a82f47a.

[4] Campos M, Cordi L, Darán N, Mei L. Antibacterial activity of chitosan solutions for wound dressing. In: Macromolecular Symposia. 2006. p. 515–8. Available from: https://www.scopus.com/inward/record.uri?eid=2-s2.0-33947181620&doi=10.1002%2fmasy.200651373&partnerID=40&md5=85e6bdac3005dd58837ccd7e1770949f.

[5] Kirkpatrick AW, Campbell MR, Jones JA, Broderick TJ, Ball CG, McBeth PB et al. Extraterrestial hemorrhage control: Terrestrial developments in technique, technology, and philosophy with applicability to traumatic hemorrhage control in long-duration spaceflight. J Am Coll Surg. 2005;200(1):64–76. Available from: https://www.scopus.com/inward/record.uri?eid=2-s2.0-13844257036&doi=10.1016%2fj.jamcollsurg2004.08.028&partnerID=40&md5=17829c3395b9a7ce0d3efdd3cba095bc. 10.1016/j.jamcollsurg.2004.08.028 15631922

[6] Özmeriç N, Özcan G, Haytaç CM, Alaaddinoǧlu EE, Sargon MF, Şenel S. Chitosan film enriched with an antioxidant agent, taurine, in fenestration defects. J Biomed Mater Res. 2000;51(3):500–3. Available from: https://www.scopus.com/inward/record.uri?eid=2-s2.0-0034609610&doi=10.1002%2f1097-4636%2820000905%2951%3a3%3c500%3a%3aAID-JBM26%3e3.0.CO%3b2-P&partnerID=40&md5=bf7b712a5783a565f483d8f8a6e77bea. 10.1002/1097-4636(20000905)51:3<500::aid-jbm26>3.0.co;2-p 10880094

[7] Giudice A, Antonelli A, Muraca D, Fortunato L (2020). Usefulness of advanced-platelet rich fibrin (A-PRF) and injectable-platelet rich fibrin (i-PRF) in the management of a massive medication-related osteonecrosis of the jaw (MRONJ): a 5-years follow-up case report. Indian J Dent Res.

[8] Di Minno A, Aveta A, Gelzo M, Tripodi L, Pandolfo SD, Crocetto F (2022). 8-Hydroxy-2-Deoxyguanosine and 8-Iso-prostaglandin F2α: putative biomarkers to assess oxidative stress damage following Robot-assisted radical prostatectomy (RARP). J Clin Med.

[9] Franco R, Gianfreda F, Miranda M, Barlattani A, Bollero P (2020). The hemostatic properties of chitosan in oral Surgery. Biomedical and Biotechnology Research Journal (BBRJ).

[10] Bollero P, ORAL HEALTH AND IMPLANT THERAPY IN PARKINSONï¿½S (2017). PATIENTS: Rev Oral Implantol (Rome).

[11] Soegiantho P, Suryawinata PG, Tran W, Kujan O, Koyi B, Khzam N, Algarves Miranda L. Survival of single immediate implants and reasons for loss: a systematic review. Prosthesis 2023;5:378–424. 10.3390/prosthesis5020028.

[12] Yokoyama M, Shiga H, Ogura S, Sano M, Komino M, Takamori H, Uesugi H, Haga K, Murakami Y. Functional differences between chewing sides of implant-supported denture wearers. Prosthesis 2023;5:346–357. 10.3390/prosthesis5020025.

[13] Vozzo LM, Azevedo L, Fernandes JCH, Fonseca P, Araújo F, Teixeira W, Fernandes GVO, Correia A. The success and complications of complete-arch implant-supported fixed monolithic zirconia restorations: a systematic review. Prosthesis 2023;5:425–436. 10.3390/prosthesis5020029.

[14] Iacono R, Mayer Y, Marenzi G, Ferreira BV, Pires GE, Migliorati M, Bagnasco F. Clinical, radiological, and aesthetic outcomes after placement of a bioactive-surfaced implant with immediate or delayed loading in the anterior maxilla: 1-year retrospective follow-up study. Prosthesis 2023;5:610–621, 10.3390/prosthesis5030043.

[15] Adel S, Zaher A, El Harouni N, Venugopal A, Premjani P, Vaid N. Robotic applications in orthodontics: changing the face of contemporary clinical care. Biomed Res Int 2021:1–16. 10.1155/2021/9954615 PMC8225419 34222490

[16] Ramli R, Ghani N, Taib H, Baharin NHM. Successful management of dentin hypersensitivity: a narrative review. Dent Med Probl. 2022;59:451–460. 10.17219/dmp/143354 36206495

[17] Kucharska M, Niekraszewicz A, Wiśniewska-Wrona M, Brzoza-Malczewska K. Dressing sponges made of chitosan and chitosan-alginate fibrids. Fibres Text East Eur. 2008;16(3):109–13. Available from: https://www.scopus.com/inward/record.uri?eid=2-s2.0-55349121202&partnerID=40&md5=7aadae52d276d10c51ff59ff905295da.

[18] Mi FL, Shyu SS, Wu YB, Lee ST, Shyong JY, Huang RN. Fabrication and characterization of a sponge-like asymmetric chitosan membrane as a wound dressing. Biomaterials. 2001;22(2):165–73. Available from: https://www.scopus.com/inward/record.uri?eid=2-s2.0-0035864265&doi=10.1016%2fS0142-9612%2800%2900167-8&partnerID=40&md5=99eebc882d9a621661414efa991c7724. 10.1016/s0142-9612(00)00167-8 11101160

[19] Hirano S. Chitin biotechnology applications. Biotechnol Annu Rev. 1996;2(C):237–58. Available from: https://www.scopus.com/inward/record.uri?eid=2-s2.0-0030309474&doi=10.1016%2fS1387-2656%2808$%2970012-7&partnerID=40&md5=19e465dba461ad487e7969d509d43a08. 10.1016/s1387-2656(08)70012-7 9704098

[20] Qazi N, Pawar M, Padhly PP, Pawar V, D’Amico C, Nicita F, Fiorillo L, Alushi A, Minervini G, Meto A. Teledentistry: evaluation of instagram posts related to bruxism. Technol Health Care. 2023;31:1923–1934. 10.3233/THC-220910. 10.3233/THC-220910 36872812

[21] Rathi S, Chaturvedi S, Abdullah S, Rajput G, Alqahtani NM, Chaturvedi M, Gurumurthy V, Saini R, Bavabeedu, SS, Minervini G. Clinical trial to assess physiology and activity of masticatory muscles of complete denture wearer following vitamin D intervention. Medicina (B Aires). 2023;59:410. 10.3390/medicina59020410. 10.3390/medicina59020410 PMC9961876 36837611

[22] Minervini G, Franco R, Marrapodi MM, Ronsivalle V, Shapira I, Cicciù M. Prevalence of temporomandibular disorders in subjects affected by Parkinson Disease: a systematic review and metanalysis. J Oral Rehabil. 2023. 10.1111/joor.13496 37183340

[23] Di Stasio D, Lauritano D, Minervini G, Paparella RS, Petruzzi M, Romano A, Candotto V, Lucchese A. Management of denture stomatitis: a narrative review. J Biol Regul Homeost Agents 2018;32:113–116. 29460527

[24] Reddy LKV, Madithati P, Narapureddy BR, Ravula SR, Vaddamanu SK, Alhamoudi, FH, Minervini G, Chaturvedi S. Perception about Health Applications (Apps) in smartphones towards telemedicine during COVID-19: a cross-sectional study. J Pers Med 2022;12: 1920. 10.3390/jpm12111920. 10.3390/jpm12111920 PMC9697835 36422096

[25] Barone S, Antonelli A, Averta F, Diodati F, Muraca D, Bennardo F (2021). Does Mandibular Gonial Angle Influence the Eruption Pattern of the Lower Third Molar? A three-dimensional study. J Clin Med.

[26] Wang X, Yan Y, Zhang R. A comparison of chitosan and collagen sponges as hemostatic dressings. J Bioact Compat Polym. 2006;21(1):39–54. Available from: https://www.scopus.com/inward/record.uri?eid=2-s2.0-31544459842&doi=10.1177%2f0883911506060201&partnerID=40&md5=a31d2774f5e11c9ef86e2a28270f7d92.

[27] Wedmore I, McManus JG, Pusateri AE, Holcomb JB. A special report on the chitosan-based hemostatic dressing: Experience in current combat operations. Journal of Trauma - Injury, Infection and Critical Care. 2006;60(3):655–8. Available from: https://www.scopus.com/inward/record.uri?eid=2-s2.0-33645549194&doi=10.1097%2f01.ta.000019939291772.44&partnerID=40&md5=1636814a21c76faf78e2a8d0927c60dd. 10.1097/01.ta.0000199392.91772.44 16531872

[28] DENTAL SUPPLEMENT, Minetti E, Palermo A, Savadori P, Barlattani A, Franco R (2019). Autologous tooth graft: a histological comparison between dentin mixed with xenograft and dentin alone grafts in socket preservation. J Biol Regul Homeost Agents.

[29] Nicolai G, Lorè B, Mariani G, Bollero P, De Marinis L, Calabrese L (2010). Central Giant Cell Granuloma of the Jaws. J Craniofac Surg.

[30] PANDEY A, KP, AVINASH A, PATHIVADA L, KUMAR B, KAPUR D. Comparative volumetric analysis of three different obturating materials in primary molars under cone beam computed tomography: an in-vitro study. Minerva Dent Oral Sci. 2023;72. 10.23736/S2724-6329.22.04679-4. 10.23736/S2724-6329.22.04679-4 36197278

[31] Antonelli A, Barone S, Bennardo F, Giudice A (2023). Three-dimensional facial swelling evaluation of pre-operative single-dose of prednisone in third molar Surgery: a split-mouth randomized controlled trial. BMC Oral Health.

[32] Morgan RL, Whaley P, Thayer KA, Schünemann HJ (2018). Identifying the PECO: a framework for formulating good questions to explore the association of environmental and other exposures with health outcomes. Environ Int.

[33] Pippi R, Santoro M, Cafolla A (2017). The Use of a Chitosan-Derived Hemostatic Agent for Postextraction Bleeding Control in patients on Antiplatelet Treatment. J Oral Maxillofac Surg.

[34] Malmquist JP, Clemens SC, Oien HJ, Wilson SL (2008). Hemostasis of oral Surgery wounds with the HemCon Dental Dressing. J Oral Maxillofac Surg.

[35] kumar J. Hemostasis and post-operative care of oral Surgical wounds by Hemcon Dental Dressing in patients on oral anticoagulant therapy: a Split Mouth Randomized Controlled Clinical Trial. Journal of Clinical and Diagnostic Research; 2016. 10.7860/JCDR/2016/17275.8462 PMC5072077 27790577

[36] Sharma S, Kale TP, Balihallimath LJ, Motimath A (2017). Evaluating effectiveness of Axiostat Hemostatic Material in achieving Hemostasis and Healing of extraction wounds in patients on oral antiplatelet Drugs. J Contemp Dent Pract.

[37] Kale TP, Singh AK, Kotrashetti SM, Kapoor A (2012). Effectiveness of Hemcon Dental Dressing versus Conventional Method of Haemostasis in 40 patients on oral antiplatelet Drugs. Sultan Qaboos Univ Med J.

[38] Radhakrishna S, Shukla V, Shetty SK (2023). Is Chitosan Dental Dressing Better Than Cotton Gauze in Achieving Hemostasis in patients on Antithrombotics?. J Oral Maxillofac Surg.

[39] Sarkar S, Prashanth NT, Shobha ES, Rangan V, Nikhila G (2019). Efficacy of platelet Rich Fibrin versus Chitosan as a hemostatic agent following dental extraction in patients on antiplatelet therapy. J Oral Biol Craniofac Res.

[40] Schreiber MA. Coagulopathy in the trauma patient. Curr Opin Crit Care. 2005;11(6):590–7. Available from: https://www.scopus.com/inward/record.uri?eid=2-s2.0-28144448490&doi=10.1097%2f01.ccx.0000186374.49320.ab&partnerID=40&md5=bb7aa4cbb75854993c20927312f1fcf6. 10.1097/01.ccx.0000186374.49320.ab 16292065

[41] Xie H, Khajanchee YS, Teach JS, Shaffer BS. Use of a chitosan-based hemostatic dressing in laparoscopic partial nephrectomy. J Biomed Mater Res B Appl Biomater. 2008;85(1):267–71. Available from: https://www.scopus.com/inward/record.uri?eid=2-s2.0-41449104089&doi=10.1002%2fjbm.b.30946&partnerID=40&md5=bb80d908ffd7c2c74ba66a069dd56820. 10.1002/jbm.b.30946 17932955

[42] Ahuja N, Ostomel TA, Rhee P, Stucky GD, Conran R, Chen Z et al. Testing of modified zeolite hemostatic dressings in a large animal model of lethal groin injury. Journal of Trauma - Injury, Infection and Critical Care. 2006;61(6):1312–20. Available from: https://www.scopus.com/inward/record.uri?eid=2-s2.0-33845726989&doi=10.1097%2f01.ta.0000240597.42420.8f&partnerID=40&md5=0feb757d1d52b71b2174bef8987ddb6b. 10.1097/01.ta.0000240597.42420.8f 17159671

[43] Stacchi C, Troiano G, Montaruli G, Mozzati M, Lamazza L, Antonelli A (2023). Changes in implant stability using different site preparation techniques: osseodensification drills versus piezoelectric Surgery. A multi-center prospective randomized controlled clinical trial. Clin Implant Dent Relat Res.

[44] Di Paola A, Tortora C, Argenziano M, Marrapodi MM, Rossi F (2022). Emerging roles of the Iron chelators in inflammation. Int J Mol Sci.

[45] Tortora C, Di Paola A, Argenziano M, Creoli M, Marrapodi MM, Cenni S (2022). Effects of CB2 receptor modulation on macrophage polarization in Pediatric Celiac Disease. Biomedicines.

[46] Shen EC, Chou TC, Gau CH, Tu HP, Chen YT, Fu E (2006). Releasing growth factors from activated human platelets after chitosan stimulation: a possible bio-material for platelet-rich plasma preparation. Clin Oral Implants Res.

[47] Cunha-Reis C, TuzlaKoglu K, Baas E, Yang Y, Haj A, El, Reis RL (2007). Influence of porosity and fibre diameter on the degradation of chitosan fibre-mesh scaffolds and cell adhesion. J Mater Sci Mater Med.

[48] Burkatovskaya M, Tegos GP, Swietlik E, Demidova TN, Castano P, Hamblin A (2006). Use of Chitosan bandage to prevent fatal Infections developing from highly contaminated wounds in mice. Biomaterials.

[49] Speechley JA, Rugman FP (1992). Some problems with anticoagulants in dental Surgery. Dent Update.

[50] Rapone B, Ferrara E, Santacroce L, Topi S, Gnoni A, Dipalma G, Mancini A, Di Domenico M, Tartaglia GM, Scarano A, et al. The gaseous ozone therapy as a promising antiseptic adjuvant of periodontal treatment: a randomized controlled clinical trial. Int J Environ Res Public Health 2022;19:985. 10.3390/ijerph19020985. 10.3390/ijerph19020985 PMC8775443 35055807

[51] Dohan Ehrenfest DM, Del Corso M, Inchingolo F, Sammartino G, Charrier JB. Platelet-rich plasma (PRP) and platelet-rich fibrin (PRF) in human cell cultures: Growth factor release and contradictory results. Oral Surgery, Oral Medicine, Oral Pathology, Oral Radiology, and Endodontology. 2010;110(4):418–21. 10.1016/j.tripleo.2010.05.059 20868991

[52] Lo Russo L, Guida L, Mariani P, Ronsivalle V, Gallo C, Cicciù M (2023). Effect of Fabrication Technology on the Accuracy of Surgical guides for Dental-Implant Surgery. Bioengineering.

[53] Spagnuolo G, Annunziata M, Rengo S. Cytotoxicity and oxidative stress caused by dental adhesive systems cured with halogen and LED lights. Clin Oral Investig. 2004;8(2). 10.1007/s00784-003-0247-y 14677051

[54] Lou CW. Process technology and properties evaluation of a chitosan-coated tencel/cotton nonwoven fabric as a wound dressing. Fibers and Polymers. 2008;9(3):286–92. Available from: https://www.scopus.com/inward/record.uri?eid=2-s2.047549091348&doi=10.1007%2fs12221-008-0046-9&partnerID=40&md5=027b28f52e51ef95ed1203be9c454322.

[55] Zhang X, Lin Z, Chen W, Song Y, Li Z. [Preparation and clinical application of polyvinyl alcohol/drug-loaded chitosan microsphere composite wound dressing]. Sheng Wu Yi Xue Gong Cheng Xue Za Zhi. 2011;28(2):381–6. Available from: https://www.scopus.com/inward/record.uri?eid=2-s2.0-84879792737&partnerID=40&md5=2ca501847e2d55c23aea98e6e2d96eef. 21604506

[56] Rodriguez-Merchan EC. Local fibrin glue and chitosan-based dressings in haemophilia surgery. Blood Coagulation and Fibrinolysis. 2012;23(6):473–6. Available from: https://www.scopus.com/inward/record.uri?eid=2-s2.0-84865477268&doi=10.1097%2fMBC.0b013e3283555379&partnerID=40&md5=fd65e2c71a5034eb2ffc72d09359b9d7. 10.1097/MBC.0b013e3283555379 22688558

[57] Schmid BC, Rezniczek GA, Rolf N, Maul H. Postpartum hemorrhage: Use of hemostatic combat gauze. Am J Obstet Gynecol. 2012;206(1):e12–3. Available from: https://www.scopus.com/inward/record.uri?eid=2-s2.0-84455208301&doi=10.1016%2fj.ajog.201109.018&partnerID=40&md5=d45379325bce18911b6f9419057c951b. 10.1016/j.ajog.2011.09.018 22011588

[58] Shanmugasundaram OL, Mahendra Gowda RV. Development and characterization of polylactic acid bandage coated with biopolymers and drugs for wound healing. Journal of the Textile Institute. 2012;103(5):508–16. Available from: https://www.scopus.com/inward/record.uri?eid=2-s2.0-84860122330&doi=10.1080%2f00405000.2011.588838&partnerID=40&md5=61088a92d30c0963f3867abd2004d791.

[59] Denyer J, Gibson E. Use of fibre dressings in children with severe epidermolysis bullosa. British Journal of Nursing. 2015;24:S38–43. Available from: https://www.scopus.com/inward/record.uri?eid=2-s2.0-84927125547&doi=10.12968%2fbjon.2015.24.Sup6.S38&partnerID=40&md5=779bd39c6d66c0ba149c0ec20c5242cf. 10.12968/bjon.2015.24.Sup6.S38 25816002

[60] Jia Tb, Chen Jy, Feng Xx, Chang J. Fabrication and characterization of chitosan/mesoporous bioactive glasses porous films. Journal of Clinical Rehabilitative Tissue Engineering Research. 2011;15(42):7877–80. Available from: https://www.scopus.com/inward/record.uri?eid=2-s2.0-84864682121&doi=10.3969%2fj.issn.1673-8225.2011.42.021&partnerID=40&md5=90c83886f055b3186b40eff1c8184864.

[61] Kranokpiraksa P, Pavcnik D, Kakizawa H, Uchida BT, Jeromel M, Keller FS et al. Hemostatic efficacy of chitosan-based bandage for closure of percutaneous arterial access sites: An experimental study in heparinized sheep model. Radiol Oncol. 2010;44(2):86–91. Available from: https://www.scopus.com/inward/record.uri?eid=2-s2.0-77953016214&doi=10.2478%2fv10019-010-0021-0&partnerID=40&md5=464cca6c7c0ae502337bbfd623a385de. 10.2478/v10019-010-0021-0 PMC3423686 22933896

[62] Pusateri AE, McCarthy SJ, Gregory KW, Harris RA, Cardenas L, McManus AT et al. Effect of a chitosan-based hemostatic dressing on blood loss and survival in a model of severe venous hemorrhage and hepatic injury in swine. Journal of Trauma. 2003;54(1):177–82. Available from: https://www.scopus.com/inward/record.uri?eid=2-s2.0-0037243875&doi=10.1097%2f00005373-200301000-00023&partnerID=40&md5=3a7d035367988f19bdfcb60355da52a7. 10.1097/00005373-200301000-00023 12544915

[63] Budala DG, Martu MA, Maftei GA, Diaconu-Popa DA, Danila V, Luchian I. The Role of Natural Compounds in Optimizing Contemporary Dental Treatment—Current Status and Future Trends. J Funct Biomater. 2023;14(5). Available from: https://www.scopus.com/inward/record.uri?eid=2-s2.0-85160254062&doi=10.3390%2fjfb14050273&partnerID=40&md5=3e536328b3a8f0475e4419dcb9c27759. 10.3390/jfb14050273 PMC10218970 37233383

[64] Kozen BG, Kircher SJ, Henao J, Godinez FS, Johnson AS. An alternative hemostatic dressing: Comparison of CELOX, HemCon, and QuikClot. Academic Emergency Medicine. 2008;15(1):74–81. Available from: https://www.scopus.com/inward/record.uri?eid=2-s2.0-44449116313&doi=10.1111%2fj.1553-2712.2007.00009.x&partnerID=40&md5=1663578c9dc24a1293d0603f8489cd86. 10.1111/j.1553-2712.2007.00009.x 18211317

[65] Alam HB, Burris D, Dacorta JA, Rhee P. Hemorrhage control in the battlefield: Role of new hemostatic agents. Mil Med. 2005;170(1):63–9. Available from: https://www.scopus.com/inward/record.uri?eid=2-s2.0-15944366571&doi=10.7205%2fMILMED.170.1.63&partnerID=40&md5=add238e683c5e5414a80a58ee3e14406. 10.7205/milmed.170.1.63 15724857

[66] Acheson EM, Kheirabadi BS, Deguzman R, Dick EJ Jr, Holcomb JB, Maxwell RA et al. Comparison of hemorrhage control agents applied to lethal extremity arterial hemorrhages in swine. Journal of Trauma - Injury, Infection and Critical Car. 2005;59(4):865–75. Available from: https://www.scopus.com/inward/record.uri?eid=2-s2.0-30044437888&doi=10.1097%2f01.ta.000018765563698.9f&partnerID=40&md5=d8789591105ed964589024c2bdc6e2db. 10.1097/01.ta.0000187655.63698.9f 16374275

